# Gestational Trophoblastic Disease: Complete versus Partial Hydatidiform Moles

**DOI:** 10.3390/diseases12070159

**Published:** 2024-07-17

**Authors:** Jeffrey Gonzalez, Meagan Popp, Stephanie Ocejo, Alvaro Abreu, Hisham F. Bahmad, Robert Poppiti

**Affiliations:** 1 Herbert Wertheim College of Medicine, Florida International University, Miami, FL 33199, USA; jgonz1074@med.fiu.edu (J.G.); mpopp002@med.fiu.edu (M.P.); socej001@med.fiu.edu (S.O.); aabre070@med.fiu.edu (A.A.); 2 The Arkadi M. Rywlin M.D. Department of Pathology and Laboratory Medicine, Mount Sinai Medical Center, Miami Beach, FL 33140, USA; robert.poppiti@msmc.com; 3 Department of Pathology, Herbert Wertheim College of Medicine, Florida International University, Miami, FL 33199, USA

**Keywords:** hydatidiform mole, gestational trophoblastic disease, complete hydatidiform mole, partial hydatidiform mole, choriocarcinoma

## Abstract

Hydatidiform moles, including both complete and partial moles, constitute a subset of gestational trophoblastic diseases characterized by abnormal fertilization resulting in villous hydrops and trophoblastic hyperplasia with or without embryonic development. This involves chromosomal abnormalities, where one or two sperms fertilize an empty oocyte (complete hydatidiform mole (CHM); mostly 46,XX) or two sperms fertilize one oocyte (partial hydatidiform mole (PHM); mostly 69,XXY). Notably, recurrent occurrences are associated with abnormal genomic imprinting of maternal effect genes such as *NLRP7* (chromosome 19q13.4) and *KHDC3L* (chromosome 6q1). Ongoing efforts to enhance identification methods have led to the identification of growth-specific markers, including p57 (cyclin-dependent kinase inhibitor 1C; *CDKN1C*), which shows intact nuclear expression in the villous cytotrophoblast and villous stromal cells in PHMs and loss of expression in CHMs. Treatment of hydatidiform moles includes dilation and curettage for uterine evacuation of the molar pregnancy followed by surveillance of human chorionic gonadotropin (HCG) levels to confirm disease resolution and rule out the development of any gestational trophoblastic neoplasia. In this review, we provide a synopsis of the existing literature on hydatidiform moles, their diagnosis, histopathologic features, and management.

## 1. Introduction

Various forms of hydatidiform moles originate from aberrantly proliferated placental tissue, predominantly comprising trophoblastic cells characterized by villous hydrops of androgenetic origin [1]. These moles manifest primarily as complete and partial moles. A complete mole results from the fertilization of an empty ovum by one or two sperms (null genome), leading to paternally-derived haploid genetic material (mainly 46,XX) [1,2]. Conversely, a partial mole arises when a viable ovum is fertilized by two or more sperms, resulting in genetic material, mainly comprising 69,XXY [1,2]. These moles exhibit distinct characteristics concerning genetic composition, associated complications, tissue consistency, and signs and symptoms in affected patients.

While a comprehensive epidemiological understanding of these moles necessitates further research, existing data indicate a higher incidence in Southeast Asia [2]. This increased incidence in Asia compared to other geographical regions may be attributed to genetic differences within ethnic groups. Geographical correlation suggests an increased incidence of molar pregnancies with advancing maternal age (>35 years) and among adolescent pregnancies. The risk significantly escalates, increasing by fivefold in women over 40 and by 2.5-fold in those over 35 [3]. Additionally, the likelihood of recurrent molar pregnancies surpasses that of experiencing a singular occurrence, along with an elevated risk of other gestational trophoblastic diseases, including choriocarcinoma [3].

A complete mole comprises solely maternally derived mitochondrial DNA, while all its nuclear DNA originates paternally. The haploid paternal genetic material undergoes duplication, resulting in a diploid 46,XX or 46,XY molar pregnancy [4]. In contrast, partial molar pregnancies feature a triploid diandric monogynic genome [2,5], typically resulting from the fertilization of an ovum by two (or more) sperms or rarely from reduplications of a single sperm’s genetic material post-fertilization, resulting in a triploid 69,XXY, 69,XXX, or 69,XYY karyotypes [6] (Table 1). Distinguishing between these entities can also occur via the expression levels of p57 observed through immunostaining. Absent or minimal expression of p57, a product of the maternally expressed *CDKN1C* gene [2,7], in the villi signifies a complete mole. Conversely, a partial mole demonstrates heightened p57 expression due to maternal chromosomal complements [2]. While immunohistochemistry has been a traditionally useful method for the identification of specific markers associated with complete and partial moles, new ancillary techniques including PCR have emerged as new options. Through the amplification of STR loci, maternal and paternal contributions to the moles can be determined while also not requiring fresh tissue. While PCR methods have become new ancillary methods in the identification of such tumors, studies have come to indicate that a comprehensive approach using both methods may allow for better diagnostic ability [8].

**Figure 1:**
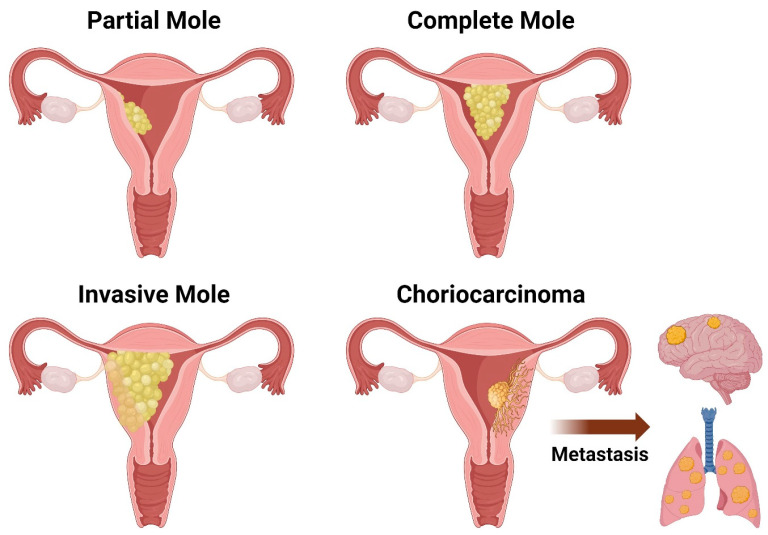
Schematic drawing of the different types of gestational trophoblastic disease (GTD): partial mole, complete mole, invasive mole, and choriocarcinoma.

The diagnosis of hydatidiform molar pregnancies hinges on the morphological, genetic, and histopathological features of the moles and can be performed through screening procedures such as array comparative genomic hybridization, which assesses genomic imbalances [12]. Clinically, women with a complete mole may display an enlarged uterus mimicking pregnancy, accompanied by markedly elevated beta-HCG levels [13]. Imaging reveals anechoic cystic spaces resembling “grape clusters” with an echogenic mass and “snowstorm” appearance [14]. In contrast, partial molar pregnancies typically do not significantly affect the uterine size; they exhibit slightly elevated beta-HCG levels and contain fetal parts, unlike complete moles, which may be visible on imaging [14] (Figure 1). Common symptoms include persistent emesis, vaginal bleeding, pelvic pressure, and pain. In any hydatidiform moles, symptoms of hyperthyroidism and thyrotoxicosis may also manifest as a result of the increased beta-HCG levels, due to the molecular mimicry between HCG and thyroid-stimulating hormone (TSH), and hence cross-reactivity with the TSH receptor, contributing to these effects [15]. Although clinical signs, symptoms, and beta-HCG levels are useful in the diagnosis of hydatidiform moles, ultrasound imaging is usually the initial screening method for a suspected diagnosis. This is due to the high frequency of ultrasounds during the first trimester during which distinct images of a complete mole can be depicted [14]. Nevertheless, the definitive diagnosis can be only made by histopathological examination of the tissue where complete moles exhibit edematous hydropic villi with circumferential and hyperplastic trophoblastic cells, while partial moles feature large hydropic villi and small fibrotic villi [2]. Further ancillary tests can also be performed to distinguish between the two entities and support the diagnosis.

Distinguishing between complete and partial moles or different gestational diseases is pivotal for tailoring sequential treatment and monitoring of patients. Recurrent molar pregnancies or gestational tumors are more prevalent in complete moles than in partial molar pregnancies, although occurrences in the latter have been documented, but recurrence in both is rare. Given the possibility of gestational tumors such as choriocarcinoma emerging post-molar pregnancy, serial follow-up measurement of beta-HCG levels is essential [2]. Of the gestational tumors, choriocarcinomas are of particular importance due to their malignant and aggressive potential [16]. Such tumors can originate from prior CHMs and PHMs when they gain metastatic potential. It is believed that the particularly aggressive nature of choriocarcinomas is associated with an uncontrolled exacerbation of the normal, controlled invasion that is part of the implantation process in the placenta. Due to the inherent capability of invading tissue, when this neoplasm becomes malignant, the extent of invasion can become extensive. To monitor gestational trophoblastic neoplasia, an HCG assay that can detect all forms of HCG, such as beta-HCG, core HCG, C-terminal HCG, nicked-free beta, beta core, and preferably the hyperglycosylated forms, should be used [17]. Specifically, with choriocarcinomas, a retrospective study determined their occurrence in 1:40,000–50,000 normal pregnancies and 1:40 hydatidiform molar pregnancies, specifically CHM [12]. Risk of gestational trophoblastic neoplasia post-CHM was as high as 25% in one study [18]. Beta-HCG levels should be monitored every 1–2 weeks until they have normalized. Once normalized, they should be monitored monthly for 6 months in CHM. However, in PHM, one more monthly normal measurement is recommended, as per the International Federation of Gynecology and Obstetrics (FIGO) recommendations [14,17].

For patients diagnosed with choriocarcinoma or other gestational trophoblastic neoplasms and receiving chemotherapy, preserving their fertility is a factor physicians should consider [19]. Preventing neoplastic sequela, along with acquiring a patient-focused history to determine the patient’s goals regarding pregnancy, may call for monitoring and preserving fertility. For women with intentions of future pregnancies, a dilatation and curettage are the primary forms of management. Through ultrasound guidance and general anesthesia, the physician can remove all the molar tissue. Due to the increased uterine size from the complete mole, there is a higher risk of excess bleeding during the procedure. Therefore, units of blood should be available, in addition to providing intravenous oxytocin to prevent blood loss. In women who no longer have goals of becoming pregnant, a hysterectomy with salpingectomy is recommended, further assisting with the prevention of gestational trophoblastic neoplasms by 80% [14].

## 2. Pathogenesis of Hydatidiform Moles

The normal development of an embryo starts with the entrance of sperm into the vaginal canal, where female reproductive mechanisms selectively guide a reduced number of sperms into the fallopian tubes. Despite the deposition of millions of sperm cells, factors such as vaginal acidity and thick cervical mucus limit the number that reaches the fallopian tubes. This process aims to facilitate the encounter between sperm and a potentially incoming oocyte after successful female ovulation. During sperm migration, capacitation occurs, activating the sperm’s ability to fertilize an oocyte. Capacitation enhances sperm mobility and facilitates penetration of the oocyte corona radiata layer by thinning the acrosome membrane. The final stage of fertilization, the acrosomal reaction, takes place in the distal uterine tube. Here, enzymes in the acrosome pierce through the corona radiata and zona pellucida, creating a path for the sperm to reach the oocyte. Upon reaching the oocyte, the sperm interacts with sperm-binding receptors, leading to the fusion of the sperm plasma membrane with that of the oocyte, allowing for embryo development [20]. Subsequent divisions result in the formation of the blastocyst, with two distinct layers—the embryoblast and the trophoblast. The embryoblast becomes the developing fetus, while the trophoblast differentiates into support structures, including syncytiotrophoblasts for invasion and HCG secretion, and cytotrophoblasts for placental formation [21,22,23]. 

However, normal fertilization and embryological development do not always lead to a developing fetus. Hydatidiform moles are due to abnormal placental development. Although rare, contributing factors, with maternal age as the primary contributor, have been identified [24,25]. Other clinically significant trophoblastic growths include invasive moles and choriocarcinoma, previously associated with poor prognoses but now showing cure rates exceeding 98% due to improved treatment protocols [25]. These tumors, associated with placental development, tend to secrete excess HCG [9].

As mentioned above, CHMs are generally diploid (46,XX) and PHMs are mostly triploid (69,XXX or 69,XXY) [25,26,27]. Understanding the clinical significance of these chromosomal abnormalities and ploidy status is crucial for predicting malignancies and complications. For example, diploid complete hydatidiform moles may progress to choriocarcinomas, while triploid partial moles rarely progress to malignancies. Patients with a history of complete hydatidiform moles face a four to five times greater risk of developing future moles than those without a prior history [28].

Hydatidiform moles can be influenced by genetic and chromosomal abnormalities, distinguishing them from other gestational trophoblastic diseases. They are unique in their potential for Mendelian inheritance, increasing the likelihood of multiple occurrences. Genetic investigations suggest that recessive maternal-effect mutations in *NLRP7* and *KHDC3L* genes, leading to abnormal methylation processes in maternally imprinted genes, can contribute to the recurrence of hydatidiform moles [29,30]. The importance of imprinting gene defects is underscored by the silencing of maternally imprinted genes while paternally imprinted versions continue to be expressed [7].

To accurately identify and classify hydatidiform moles, p57 protein (encoded by *NLRP7* gene) expression has been identified as a valuable supporting marker, complementing genetic assessments. Approximately 50 mutations of the *NLRP7* gene, and around 10–14% involving *KHDC3L*, are implicated in the development of hydatidiform moles [7]. Understanding the functions of these genes reveals their involvement in oocyte growth regulation, imprint establishment initiation, juxta perinuclear signals display, and colocalization within the oocyte [27]. Imprinting gene defects is crucial, as they lead to the silencing of maternally imprinted genes while allowing the continued expression of paternally imprinted versions [7]. Further research into these developmental patterns enhances our comprehension of hydatidiform moles and their underlying genetic mechanisms [2,30].

## 3. Histopathological Features of Hydatidiform Moles

Hydatidiform moles constitute a subset of gestational trophoblastic diseases characterized by abnormal placental tissue, presenting histological changes in chorionic villi and trophoblast cells [10]. It is important to identify those changes and features and rule out higher-grade diseases or neoplasms such as persistent trophoblastic disease (invasive mole) and choriocarcinoma [10]. Grossly, both CHMs and PHMs are capable of causing uterine cavity expansion due to friable and transparent cystic masses [10] (Figure 1). Histologically, both exhibit cystic edematous chorionic villi swelling and trophoblastic hyperplasia, limiting the recognition of vascular structures on microscopic examination [11]. Nevertheless, there are some unique features that enable us to distinguish between the two.

### 3.1. Complete Hydatidiform Moles (CHM)

Gross examination of CHMs reveals hydropic semitransparent chorionic villi and circumferential trophoblastic proliferation. Advanced forms may exhibit a honeycomb appearance. Microscopically, poorly vascularized chorionic villi with hydropic swelling and myxomatous, edematous stroma creates a florid cistern filled with stromal fluid. The polypoid appearance may be evident early in the first trimester. Defective pericyte recruitment to stromal vessels in CHMs, as observed in studies, may contribute to hydropic changes [9,10,31,32]. The absence of embryonic tissue results from early tissue death before a functioning circulation system is established [31]. Increased trophoblastic proliferation is linked to elevated endothelial growth factor receptor (EGFR) expression in trophoblastic cells. The absence of p57 expression due to the androgenic origin of CHMs explains the increased trophoblastic and stromal proliferation and invasive potential. Compared to partial moles, CHMs have a higher risk of developing both an invasive mole and choriocarcinoma.

### 3.2. Partial Hydatidiform Moles (PHM)

Compatible with early embryogenesis, PHMs may exhibit fetal parts by imaging or gross examination. Less trophoblastic proliferation, focal distribution of proliferative areas, and edematous villi in only some regions distinguish PHMs from CHMs. Histologically, villous trophoblastic inclusions may be seen. The slower rate of change from normal placental tissue to a hydatidiform morphology explains these characteristics. Microscopic examination may resemble CHMs but with less marked changes and a focal distribution throughout the tissue [9]. Due to challenges in differentiation through imaging, ancillary studies and karyotyping may be necessary. Unlike CHMs, PHMs exhibit positive p57 nuclear staining due to the presence of maternal genes [9]. Understanding these histological and genetic differences between CHMs and PHMs is crucial for accurate diagnosis and appropriate management.

## 4. Ancillary Studies: Immunohistochemical Markers

Initial histopathological diagnosis of hydatidiform moles may be made by identification of aspects of villous dysmorphism and abnormal villous trophoblast hyperplasia; however, the distinction of PMH and CMH is aided by ancillary immunohistochemistry techniques, most of which are qualitative by assessing for the presence of immunopositivity rather than quantifying protein expression [33]. Especially in the cases of early CHM, the diagnosis can be quite challenging and often necessitates additional staining methods [34]. Given the restrictions of morphological assessment and the pivotal clinical significance of precisely diagnosing molar specimens, it is strongly advised to employ supplemental techniques to enhance the accuracy of hydatidiform mole diagnosis.

### 4.1. p57

P57 is a cyclin-dependent kinase inhibitor protein and is the product of the paternally imprinted, maternally expressed gene *CDK1C* (*p57KIP2*) located on chromosome 11p15.5 [35]. Due to the gene being maternally expressed, p57 expression is lost in CHMs and is retained and visualized as a nuclear stain in PHMs and non-molar specimens [2,36,37,38,39]. It is important to acknowledge that in rare instances, mosaic androgenetic/biparental gestations and twin (complete mole and non-molar) gestations may demonstrate discordant p57 staining patterns, inconsistent with the morphology [40,41]. However, overall, despite these exceptions, p57 remains a convenient and cost-effective method to support a diagnosis of PHM or non-molar gestations [42,43].

Vang et al. analyzed 80 genotyped cases from a series of 200 potential molar specimens. The inclusion of the p57 immunostain enhanced the sensitivity of diagnosing CHMs, increasing the range from 93% to 96% for individual pathologists and reaching 96% by consensus. Moreover, it improved specificity, raising the range from 96% to 98% for individual pathologists and maintaining 96% by consensus. However, the addition of the p57 stain showed no substantial influence on the diagnosis of PHM and non-molar pregnancies [44].

In a study performed by Landolsi et al., immunohistochemical expression of p57 protein was investigated in 220 first-trimester hydropic abortus specimens and compared to original diagnoses based on morphological characteristics. Among the samples, there were 132 CHM, 49 PHM, and 39 cases of hydropic abortion (HA). In total, 210 of the cases revealed concordant p57 staining results. Among the 10 cases showing discordant diagnoses, there was negative p57 staining in PHMs or HAs [38]. Therefore, while p57 can be used successfully to distinguish CHM from its mimics, other molecular techniques such as DNA microsatellite genotyping may be used in challenging cases with discordant positive p57 staining.

### 4.2. Twist 1

Another potential immunohistochemical marker is Twist 1, a protein involved in placental formation that induces epithelial-mesenchymal transitions (EMT) and trophoblastic differentiation and invasion [45]. The process of EMT necessitates the reduction in cell junction proteins, among which the cadherin family is downregulated [46]. Cadherins are mediators of cell-to-cell adhesions in epithelial tissues and regulators of trophoblast cell behavior in placental development [46]. Not only does Twist 1 downregulate cadherins but it also activates the transcription of genes characteristic of a mesenchymal state [45].

Jahanbin et al. conducted a cross-sectional study in which 47 cases of CHM and 40 cases of PHM were randomly chosen using histopathological criteria. Only the cases that were mutually confirmed by two expert gynecological pathologists and validated through p57 immunohistochemistry study were included. The expression of the Twist-1 marker in villi stromal cells, as well as syncytiotrophoblasts, was evaluated quantitatively (percentage of positive cells), qualitatively (staining intensity), and as a total comprehensive score. Expression of Twist-1 was found to be higher and more intense in villous stromal cells of CHMs (*p* < 0.001). The study suggested that moderate to strong staining intensity in more than 50% of villous stromal cells can differentiate CHM and PHM with 89.5% sensitivity and 75% specificity. In syncytiotrophoblasts of CHMs, Twist-1 expression was significantly lower than PHMs (*p* < 0.001). Also, the study concluded that negative or weak staining intensity in less than 10% of syncytiotrophoblasts, can distinguish CHM and PHM with 82.9% sensitivity and 60% specificity [47]. Therefore, a higher expression of Twist-1 in villous stromal cells of hydatidiform moles is a sensitive and specific marker for the diagnosis of CHMs.

Furthermore, Moussa et al. analyzed Twist 1 expression in 55 cases of CHM, PHM and HA. The study reported that Twist1 expression can distinguish CHM from PHM and HA with 100% sensitivity, 100%, specificity, 100% positive predictive value (PPV), and 100% negative predictive value (NPV). The study also assessed the combined Ki-67 and E-cadherin expression showing that it could differentiate PHM and HA with 100% sensitivity, 93.3% specificity, 92.3% PPV, and 100% NPV [42].

### 4.3. Ki-67

Ki-67 protein is another protein that is crucial in the process of trophoblast differentiation and can be visualized as a nuclear stain in cytotrophoblasts and intermediate trophoblasts to differentiate CHM [42]. The protein is typically expressed throughout all active phases of the cell cycle (G1, S, G2 and mitosis) [48]. The lack of Ki67 protein expression in resting cells indicates that this protein serves as an excellent marker reflecting tissue propagation and the presence of CHM [48].

In addition to Twist-1, Moussa et al. analyzed the Ki-67 expression of their 55 cases of CHM, PHM, and HA, and found that in CHMs, the mean ± SD Ki-67 positivity was 60.6 ± 20.2% with a range of 30% to 90%. In PHM cases, the range of Ki-67 positivity was 10% to 50%, with a mean ± SD of 24.1 ± 11.8% [42].

In a study by Zhao et al., a total of 337 patient specimens classified as hydatidiform moles were analyzed for Ki-67 reactivity. Ki-67 was found in 163 of the 188 cases of CHM, compared with 103 of the 149 cases of PHM, resulting in an OR of 3.28 (*p* < 0.0001). Their results indicated that immunohistochemical assessment of Ki-67 may be a helpful and simple method to differentiate CHM from PHM [49].

Additionally, Khooei et al. reported the usefulness of Ki-67 expression in differentiating CHM and PHM from HA by performing immunohistochemical staining on 19 molar (8 PHM and 11 CHM) and 10 non-molar hydropic abortions. They found that positive cells were found to be mostly villous cytotrophoblasts and there was a significant difference in Ki-67 expression between HA and CHM (*p* < 0.001), HA and partial moles (*p* = 0.002), and also between complete and partial moles (*p* < 0.001) [50].

### 4.4. E-Cadherin

Cadherins, as mentioned above, play a crucial role as mediators of cell-to-cell adhesion within epithelial tissues and act as regulators of trophoblast cell behavior during placental development [46]. The expression of E-cadherin, seen on the villous cytotrophoblast cell membrane, serves as a criterion for the positivity of PHM [42]. Moussa et al. analyzed the expression of E-cadherin in hydatidiform moles and quantified it as the E-cadherin immunoreactivity score (EIRS), which was calculated by multiplying the percentage of positive cells by the staining intensity. EIRS expression was significantly higher in PHM compared to CHM (*p* < 0.001) and significantly higher in HAs compared to PHM (*p* < 0.001). In summary, Moussa et al. reported Twist1 expression as a highly reliable marker for the diagnosis of CHM, whereas combined Ki-67 and E-cadherin immunoreactivity can distinguish PHM from non-molar pregnancies.

Erol et al. also noted E-cadherin expression specifically on the cell membrane of villous cytotrophoblasts. The expression pattern was markedly elevated in HAs compared to PHMs and CHMs with the expression in PHMs significantly higher than CHMs (*p* < 0.01) [51].

### 4.5. p53

P53 is a tumor suppressor gene that ensures that cells repair any damaged DNA before cell division by inducing cell arrest and allowing time for DNA repair and is mostly found in the nucleus of villous cytotrophoblasts [51,52]. Missaoui et al. reported that p53 expression was higher in CHM (73.6%) compared to PHM (24.4%, *p* < 0.0001) and HA (12.8%, *p* < 0.0001) suggesting its potential utility in distinguishing CHM from PHM and HA [53].

Chen et al. also found significantly increased p53 expression in CHM (85%, 17/20) compared to PHM (60.9%, 14/23) (*p* < 0.05). Additionally, they reported significantly higher expression in PHM than in HA [54]. Kheradmand et al. reported a similar finding as p53 was positive in 60% of patients with PHM and 25% of patients with hydropic pregnancy (*p* = 0.027) with PHM specimens having a significantly higher grade of staining (*p* < 0.001) [55].

Erol et al. also found significantly increased p53 expression in CHM compared to HA and PHMs (*p* < 0.001) but no significant difference between HA and PHMs (*p* > 0.017). In summary, throughout the literature, p53 is consistently found to have increased expression in CHM compared to PHM.

### 4.6. p63

P63 is a transcription factor and a member of the p53 gene family that regulates epidermal keratinocyte proliferation and embryonic epidermal growth [56]. By analyzing nuclear immunoreactivity, Heidarpour et al. found that p63 could serve as a helpful marker in distinguishing molar from non-molar pregnancy and PHM from CHM as reactivity was higher in PHMs. However, they acknowledged the limitation that p63 is not a suitable diagnostic test on its own due to its low sensitivity and specificity. They recommended p63 as an adjuvant test to Ki-67 [57].

Furthermore, Ramalho et al. reported that there was no difference in the distribution of p63 positive cells between HA, PHM, and CHM; however, the intensity of p63 expression was stronger in PHM and CHM than HA. This finding proved p63 to be potentially useful in distinguishing hydatidiform moles from HA but not in differentiating PHM from CHM. They also stated that p63 may be useful to differentiate choriocarcinoma from other gestational trophoblastic disease [58].

Additionally, a study by Missaoui et al. reported that p63 immunoexpression was significantly higher in CHM (85.7%) and PHM (78%) compared with HA(10.2%, *p* < 0.0001 and *p* = 0.0001, respectively), further indicating p63’s utility in distinguishing hydatidiform moles from its mimics [53].

### 4.7. BCL2

Finally, BCL-2 is an anti-apoptotic protein that is mainly localized to the inner mitochondrial membrane and nuclear envelope [59]. Missaoui et al. found that BCL-2 immunostaining was significantly higher in PHM (61%) and CHM (70.7%) compared to HA (7.7% *p* = 0.001, *p* < 0.0001, respectively) [53]. Furthermore, Al Jabri et al. reported significant difference in BCL-2 intensity between PHM and CHM (*p* = 0.0005), indicating that CHM have decreased BCL-2 expression compared to PHM and normal trophoblasts, further indicating increased apoptosis and uncontrolled trophoblastic proliferation [60].

### 4.8. Differential Immunohistochemical Expression in CHMs versus PHMs

Overall, various immunohistochemical methods may be used to distinguish PHM from CHM. In summary, markers specific to CHM include negative p57 expression and positive expression of Twist 1, Ki-67, and p53 while markers specific to PHM include positive expression of p57. E-cadherin and BCL-2 are usually positive in hydatidiform moles compared to non-molar hydropic abortions. All these markers can be valuable in supporting the diagnosis of PHM and CHM and help in differentiating them from non-molar abortions.

## 5. Conclusions and Future Directions

This review offers insights into the pathogenesis, histopathological characteristics, and distinctive immunohistochemical markers associated with hydatidiform moles. Although distinguishing PHM and CHM based solely on histology may pose challenges, the use of immunohistochemical markers proves invaluable in distinguishing between the two diagnoses, facilitating a more accurate prognosis prediction.

Early diagnosis of hydatidiform moles is crucial given the potential for increased medical complications over time. However, early detection can be challenging due to non-specific ultrasound findings in preliminary presentations. Key sonographic features are often less pronounced or even absent in early stages of hydatidiform mole development [61]. Sparse hydropic change and rare cistern formation are just two detection challenges that sonographers face when presented with an early hydatidiform mole case [62]. Although histological examination remains the gold standard for hydatidiform mole detection, there is room for improvement to enhance sonographers’ confidence in investigating hydatidiform moles.

Savage et al. highlighted that CHM is comparatively easier to detect due to its distinctive snowstorm appearance. In contrast, diagnosing a PHM remains a challenge due to its diverse range of ultrasound presentations. While some progress has been made in improving the ultrasound detection of PHM, the detection rates do not align with its significantly higher prevalence compared to CHM [63].

Moreover, molecular genotyping can significantly assist in the histochemical diagnosis of HM. Traditional cytogenetics (karyotyping), DNA ploidy analysis (flow cytometry, image analysis), and fluorescence in situ hybridization (FISH) encounter a limitation in distinguishing maternal and paternal contributions; thus, they lack the generation of a specific genetics-based diagnosis. While these techniques can yield diploid and triploid results, they fail to differentiate between CHM and non-molar gestations. In contrast, short tandem repeat (STR) genetic analysis has provided a strong alternative to hydatidiform mole diagnosis. STRs are repetitive DNA sequences that are highly polymorphic in the population and were developed for identity, forensic, criminal, and relationship testing and involve PCR amplification. In this technique, alleles in villous tissue are identified as paternal or maternal, and the copy number/dosage of each allele contributes valuable diagnostic information. However, challenges may arise with mosaic forms of hydatidiform moles, such as rare triandric tetraploid examples, which could be erroneously interpreted as triploidy if a sufficient number of informative loci are not evaluated. Given the promising outcomes from the genetic analysis of hydatidiform moles, further research on the application of STR analysis and training for successful specimen collection should be pursued. This approach holds the potential to provide accurate genotyping and differentiate between PHM and CHM effectively [2]. Single nucleotide polymorphism (SNP) array also plays a role in this context. The development of high-density SNP arrays for genotyping has enabled large-scale SNP studies and comprehensive analysis to determine chromosome parental origin [64].

The timely and effective utilization of these diagnostic tools is crucial to prevent serious sequela, including choriocarcinoma, invasive mole, and potential loss of fertility. Notably, for stage I choriocarcinoma, a single-agent chemotherapy cured 83% of patients, with the other 1% achieving remission with additional chemotherapy or surgery. Stage II-IV was cured with surgery and additional chemotherapy [65]. In the case of invasive mole, chemotherapy is highly effective, but it may necessitate a hysterectomy, leading to loss of fertility [66]. Consequently, for many women, the primary concern following an hydatidiform mole diagnosis is the potential impact on their fertility. Fortunately, the preferred treatment for a hydatidiform mole is typically dilation and curettage, with a hysterectomy reserved only for cases where the patient does not desire future pregnancies or in the presence of life-threatening bleeding. Moreover, as most choriocarcinomas and invasive moles respond well to single-agent therapy, studies have reported a fertility rate of 86.7% among patients wishing to conceive after treatment [67]. Therefore, it is paramount to thoroughly discuss treatment options and the patient’s prognosis, ensuring they have a comprehensive understanding of their diagnosis. Achieving accurate information dissemination relies on the successful collection of specimens and histopathologic diagnostics. Consequently, significant care and effort should be directed toward continuing to make accurate diagnoses and advance diagnostic techniques.

## Figures and Tables

**Table 1:** Complete hydatidiform mole versus partial hydatidiform mole [9,10,11].

Feature	Complete Hydatidiform Mole	Partial Hydatidiform Mole
Embryogenesis	Not compatible with embryogenesis	Compatible with embryogenesis
Fetal tissue	Rarely contains fetal tissue	Contains fetal tissue
Common karyotype	Diploid 46, XX	Triploid 69, XXY
Genetic material	Only paternal genetic material present	Both maternal and paternal genetic material present
Fertilization	Ovum without genetic material is fertilized by two sperm or a diploid sperm cell	Haploid ovum fertilized by two sperm cells or a diploid sperm
Uterine anatomy	Uterine cavity is expanded by a friable and clear cystic mass	Uterine cavity is expanded by a friable and clear cystic mass
Gross	Hydropic semitransparent villi of various sizes Absence of normal placental tissue Grossly, early CHM may have normal-appearing villi Advanced CHM may have a honeycomb appearance Honeycomb appearance rarely seen in the first trimester	Fetal parts may be seen on imaging and/or gross examination Grossly, PHM may have normal-appearing villi
Histology	Florid cistern formation, diffuse and circumferential trophoblastic proliferation, and absence of fetal parts Hydropic swelling of poorly vascularized chorionic villi Cytological atypia and mitotic figures may be present In the first trimester, villi may have a polypoid morphology with abnormal villous stromal changes and some degree of trophoblastic hyperplasia	Some of the chorionic villi may appear normal Edematous state is appreciable on only some of the villi Slight trophoblastic proliferation with a focal distribution Histological features may be similar to CHM but less marked
Nuclear p57	Negative p57 nuclear staining due to absent expression of the gene in cytotrophoblast and stoma due to androgenic origin	Positive p57 nuclear staining due to maternal genes present
Invasive potential	More invasive potential due to androgenic origin	Less invasive potential
Risk of invasive mole and choriocarcinoma	Increased risk	No increased risk

## Data Availability

No new data were created or analyzed in this study. Data sharing is not applicable to this article.
